# The Prognostic Value of a Geriatric Risk Score for Older Patients with Colorectal Cancer

**DOI:** 10.1245/s10434-018-6867-x

**Published:** 2018-10-25

**Authors:** E. T. D. Souwer, D. Hultink, E. Bastiaannet, M. E. Hamaker, A. Schiphorst, A. Pronk, J. M. van der Bol, W. H. Steup, J. W. T. Dekker, J. E. A. Portielje, F. van den Bos

**Affiliations:** 1 Department of Internal Medicine, Haga Hospital, PO Box 40551, 2504 LN The Hague, The Netherlands; 2 Department of Medical Oncology, Leiden University Medical Center, Leiden, The Netherlands; 3 Department of Geriatrics, Diakonessenhuis, Utrecht, The Netherlands; 4 Department of Surgery, Diakonessenhuis, Utrecht, The Netherlands; 5 Department of Geriatrics, Reinier de Graaf Gasthuis, Delft, The Netherlands; 6 Department of Surgery, Haga Hospital, The Hague, The Netherlands; 7 Department of Surgery, Reinier de Graaf Gasthuis, Delft, The Netherlands; 8 Department of Geriatrics, University Medical Center Utrecht, Utrecht, The Netherlands; 9 Department of Surgery, Leiden University Medical Center, Leiden, The Netherlands

## Abstract

**Introduction:**

VMS is a Dutch risk assessment tool for hospitalized older adults that includes a short evaluation of four geriatric domains: risk for delirium, risk for undernutrition, risk for physical impairments, and fall risk. We investigated whether the information derived from this tool has prognostic value for outcomes of colorectal surgery.

**Methods:**

All consecutive patients over age 70 years who underwent elective colorectal cancer surgery in three Dutch hospitals (2014–2016) were studied. The presence of risk was scored prior to surgery and per geriatric domain as either 0 (risk absent) or 1 (risk present). The total number of geriatric risk factors was summed. The primary outcome was long-term survival. Secondary outcomes were postoperative complications, including delirium. Cox proportional hazards models were used to evaluate the sumscore and risk factors associated with overall survival.

**Results:**

Five hundred fifty patients were included. Median age was 76.5 years, and median follow-up was 870 days. Patients with intermediate (1–2) or high (3–4) sumscore were independently associated with lower overall survival, with hazard ratio (HR) of 1.9 [95% confidence interval (CI) 1.1–3.5; *p* = 0.03] and 8.7 (95% CI 4.0–19.2; *p* < 0.001), respectively. Sumscores were also associated with postoperative complications (intermediate sumscore OR 1.8; 95% CI 1.2–2.7; high sumscore OR 2.4; 95% CI 1.02–5.5).

**Conclusions:**

This easy-to-use geriatric sumscore has strong associations with long-term outcome and morbidity after colorectal cancer surgery. This information may be included in risk models for morbidity and mortality and can be used in shared decision-making.

In Europe, colorectal cancer is the second most common cancer in women and the third most common in men.^1^. Colorectal cancer is an age-related disease; over 50% of all newly diagnosed patients are 70 years or older.^2^ Older patients represent a heterogeneous population due to differences in comorbidity, functional capacity, and presence of geriatric impairments. These impairments can lead to decreased physiological reserves and diminished resistance to stressors and increase the risk of adverse outcomes of treatment.^3^ Not only do older patients have a fourfold higher risk of adverse postoperative outcomes,^4^ but they are also more likely to experience a postoperative decline in physical function resulting in functional dependency and decreased quality of life.^5^

Geriatric assessment (GA) can be used to detect previous unaddressed problems in older patients. Information derived from GA can be used to discuss treatment options and improve functional status and possibly survival.^6^ However prognostic information for patients with geriatric impairments is scarce, and currently available risk prediction tools for electively operated colorectal cancer patients do not include geriatric parameters.^7^–^9^ Therefore, more prognostic information is required for the challenging process of shared decision-making in older patients.

In The Netherlands, for all older hospitalized patients over 70 years, standard care at admission includes a short evaluation of four important geriatric domains: risk for undernutrition, physical impairment, risk for delirium, and fall risk, independently of whether GA is performed. This screening tool was implemented nationwide in 2012 as part of a National Patient Safety Program (VMS) after studying adverse events and potentially preventable deaths in Dutch hospitals and to direct geriatric interventions. Although VMS does not replace GA, this easy-to-use and well-implemented geriatric tool could provide useful prognostic information, as it is also performed for all patients prior to elective surgery.

In this study, we investigated whether a cumulative risk score composed of undernutrition, physical impairment, risk for delirium, and fall risk has prognostic value for survival and complications independently of age and American Society of Anesthesiologists (ASA) score in a large cohort of older electively operated colorectal cancer patients.

## Patients and Methods

### Study Population

All patients aged 70 years or older with surgical treatment for colorectal cancer between 1 January 2014 and 31 December 2016 in three teaching hospitals in The Netherlands (Haga Hospital in The Hague, Diakonessenhuis Hospital in Utrecht, and Reinier de Graaf Gasthuis Hospital in Delft) were included in this cohort study. Patients with acute or urgent surgery, transanal endoscopic microsurgery (TEM), stage IV colorectal carcinoma, or a synchronous second malignancy were excluded.

The primary outcome for this study was overall survival. Secondary outcomes were postoperative complications (surgical, cardiopulmonary, delirium, and other complications), readmission within 30 days, and (temporary) discharge to a rehabilitation center or nursing home.

### Data Collection

Preoperative patient characteristics and surgical outcome parameters were retrieved from the prospectively collected Dutch Colorectal Audit (DCRA). We complemented this with data from electronic medical records (EMR) for geriatric parameters. Follow-up on survival status was available until 1 February 2018 through linkage with the Municipal Personal Records Database.

From the DCRA, we retrieved the following data: age, gender, ASA score, comorbidity, and oncological data (i.e., tumor type, tumor location, and staging), surgical approach (open or laparoscopic), type of surgery (acute, urgent or elective), postoperative complications, hospital stay, readmissions within 30 days, and 30-day mortality. The Charlson comorbidity index (CCI)^10^ was calculated for all patients. Postoperative complications registered in this audit were subdivided into surgical complications, cardiopulmonary complications, and other complications. The number of total complications refers to the number of patients with one or more complications. When two or more surgical complications occurred, the most severe surgical complication was registered. Surgical complications included wound infections, bleeding, ileus, and complications that needed intervention (including anastomotic leaks). Cardiopulmonary complications consisted of pulmonary complications (pneumonia, atelectasis, pulmonary embolism, pulmonary insufficiency, or other pulmonary complications) and cardiac complications (myocardial infarction, heart failure, arrhythmia, angina pectoris, cardiac arrest, or other cardiac complications). Other complications consisted of infectious complications, thromboembolic complications, and complications defined as other in the DCRA.

Presence of delirium after surgery as well destination after discharge (to home or an extended care facility) could not be retrieved from the DCRA and was also extracted from the EMR. Delirium was defined as present when (1) the occurrence was documented in a patient’s medical record by a geriatrician or treating physician, (2) haloperidol was prescribed during hospital stay or (3) Delirium Observation Screening Scale^11^ ≥ 3 in three consecutive moments was recorded in the medical record.

### Geriatric Parameters (Used in VMS)

In the participating hospitals, the risk for undernutrition (or at risk of becoming undernourished), physical impairment, risk of delirium, and fall risk were assessed preoperatively by nursing staff with screening questionnaires. The full 13-item list of the four questionnaires is presented in “Appendix 1” (Electronic Supplementary Information).

Risk for undernutrition was assessed using either the Short Nutritional Assessment Questionnaire (SNAQ)^12^ or Malnutrition Universal Screening Tool (MUST).^13^ Increased risk for undernutrition was defined as SNAQ score ≥ 3 or MUST score ≥ 2. Functional impairment was assessed with the six-item Katz activities of daily living (ADL),^14^ consisting of questions regarding bathing, dressing, using the toilet, eating, transferring from bed to chair, and if they used incontinence materials. An impaired score was defined as Katz ADL score ≥ 2. Fall risk consisted of one question and was either present or absent. Risk for delirium was assessed using three yes or no questions scoring 1 or 0. Score ≥ 1 was considered as an increased risk. We kept the cutoff value of 1 for the delirium score as suggested by the national guidelines, as Heim et al. earlier showed its independent association with increased care, worse ADL functioning, and short-term mortality in an unselected group of older hospitalized patients.^15^

For this study, we composed a cumulative risk score of the VMS by summing the total number of impairments. All individual domains were included, independent of whether the individual domain was significantly associated with the outcomes. Low risk score was defined as sumscore of 0, intermediate risk as sumscore of 1 or 2, and high risk as sumscore of 3 or 4.

In all three hospitals, geriatric information is registered in the EMR prior to surgery for the majority of patients on the day of admission, except for malnutrition, where screening is done shortly after the decision for surgery is made. The information from the VMS does not alter the primary therapeutic plan but is used to guide supportive measures after surgery. For patients with impairments in the individual domains of falls and ADL dependency, this is postoperative mobilization with physiotherapy. For patients who have undernutrition, dietary support is advised, and in case of increased risk for delirium, postoperative monitoring using Delirium Observation Screening Scale^11^ is advised.

### Statistical Analysis

We used descriptive analysis expressing normally distributed variables as mean with standard deviation (SD) and nonnormally distributed variables as median with interquartile range (IQR). Frequencies are presented as number and percentage. A Chi squared test was used to compare proportions between the three risk groups.

To assess the prognostic value of the three risk scores on overall survival (OS), a multivariate Cox proportional hazards model was used to estimate hazard ratios (HRs) with corresponding 95% confidence interval (CI). To assess the association between risk factors and postoperative outcomes, multivariate logistic regression models were used to calculate odds ratios (ORs) with corresponding 95% CI. Age, male gender, and tumor stage were considered potential confounders and were added to a multivariate model in addition to ASA score. All analyses were performed using SPSS version 24.0 (SPSS, Inc., Chicago, IL). *p*-Values < 0.05 were considered statistically significant.

## Results

A total of 707 patients aged 70 years or older were identified. After excluding 157 patients based on the predefined criteria, 550 patients were included in the analysis, of whom 293 (53%) were men.

The median age was 76.5 years (IQR 74.3–82.1 years). Median follow-up was 870 days, and 60 deaths (11%) were registered. Table 1 presents the demographic characteristics and geriatric parameters. Most patients had laparoscopic surgery (*n* = 436; 79%). Thirty-five patients (6%) had Katz ADL score of 2 or higher, and at least one fall in the past 6 months was reported by 76 patients (14%) prior to surgery. For 27 patients (5%), the fall history was unknown. Undernutrition was present in 127 patients (23%), and 106 patients (19%) were at risk for delirium. A total of 303 patients (55%) had low risk sumscore, 220 patients (40%) had intermediate risk sumscore, and 27 patients (5%) had high risk sumscore.

**Table 1:** Patient characteristics at baseline

Patient characteristic	Total patients (*N* = 550)
Median (IQR) age (years)	76.5 (74.3–82.1)
Gender	
Male	293 (53)
Female	257 (47)
ASA score	
III–IV	172 (31)
CCI ≥ 2	211 (38)
Tumor location	
Colon	432 (79)
Rectum	118 (21)
Surgical approach	
Laparoscopic	436 (79)
Tumor stage	
I	167 (30)
II	214 (39)
III	169 (31)
Geriatric characteristics	
Katz-ADL ≥ 2	35 (6)
Fall risk	76 (14)
Risk for undernutrition	106 (19)
Risk for delirium	119 (22)

Values expressed as number (%)

Median age expressed as mean with interquartile range (IQR)

*ASA* American Society of Anesthesiologists; *CCI* Charlson comorbidity index

### Primary Outcome

**Fig. 1:**
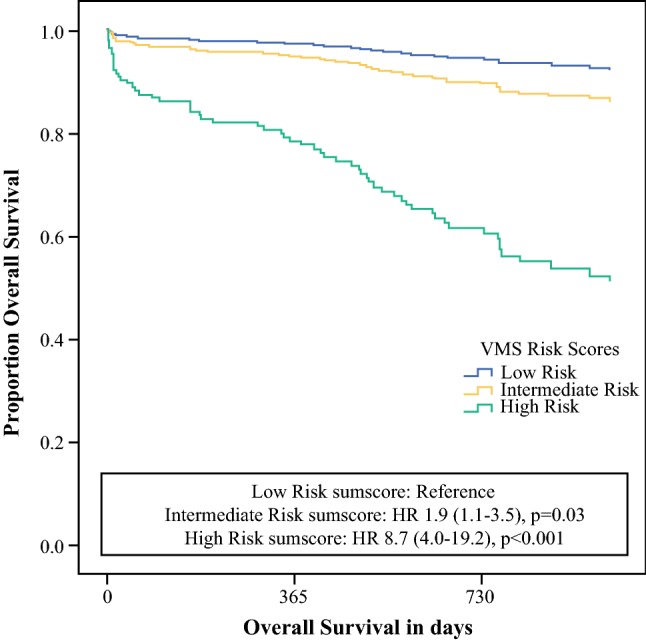
Overall survival stratified by the three risk scores

Twenty-five patients (5%) died within 6 months after surgery, and 31 patients (6%) died within 1 year. Figure 1 presents survival analysis for all three risk groups. Patients with intermediate risk sumscore (HR 1.9; 95% CI 1.1–3.5; *p* = 0.03) and high risk sum score (HR 8.7; 95% CI 4.0–19.2; *p* < 0.001) had significantly lower overall survival. At end of follow-up, 13% (*n* = 29) in the intermediate risk group and 44% (*n* = 12) in the high risk group were deceased compared with 6% (*n* = 19) in the low risk group.

When analyzing the individual domains separately and corrected for age, gender, tumor stage, and ASA score, we found that impaired functionality (Katz ≥ 2), fall risk, and risk for delirium were all associated with overall survival with HR of 4.7 (95% CI 2.5–8.8), 2.6 (95% CI 1.4–4.6), and 3.5 (95% CI 2.0–6.0), respectively. Risk for undernutrition was not independently associated with overall survival (Table 2).

**Table 2:** Surival analysis for the individual geriatric domains

	HR (95% CI)	*p*-value
Katz ADL ≥ 2	4.7 (2.5–8.8)	< 0.001
Fall risk	2.6 (1.4–4.6)	0.002
Risk for malnutrition	1.5 (0.8–2.7)	0.2
Risk for delirium	3.5 (2.0–6.0)	< 0.001

Risk scores corrected for age, gender, tumor stage, and ASA score

### Secondary Outcomes

One hundred ninety-one patients (35%) had one or more complications: 16% had surgical complications (*n* = 87), 9% cardiopulmonary complications (*n *= 48), and 6% suffered from delirium (*n* = 34). Mean length of stay was 8.7 days (± 7.0 days standard deviation, SD). Forty-eight patients (9%) were readmitted within 30 days after discharge, and 98 patients (18%) were discharged to a nursing home or rehabilitation center. The complication rate (any complication) was 28.7% in the low risk group, 40.9% in the intermediate risk score group, and 51.9% in the high risk group. See “Appendix 2” for detailed information on complications and outcomes.

In the multivariate model, intermediate risk sumscore (OR 1.8; 95% CI 1.2–2.7, *p* = 0.003) and high risk sumscore (OR 2.4; 95% CI 1.0–5.5, *p* = 0.04) were both associated with complications. Intermediate risk score was also independently associated with delirium (OR 2.9; 95% CI 1.3–6.4, *p* = 0.009) and discharge not to home (OR 2.7; 95% CI 1.6–4.5, *p* < 0.001).

We could not find an independent association between the risk sumscore and surgical complications, cardiopulmonary complications, or readmission.

Analysis of the individual domains of the risk scores showed that Katz ADL score ≥ 2 (OR 3.5; 95% CI 1.6–7.3) and risk for delirium (OR 1.4; 95% CI 1.0–1.9) were independently associated with complications. Katz ADL ≥ 2 was also independently associated with discharge not to home (OR 2.9; 95% CI 1.4–6.3) and readmission (OR 2.9; 95% CI 1.4–6.3). Risk for delirium was independently associated with delirium (OR 2.1; 95% CI 1.4–3.1), discharge not to home (OR 1.8; 95% CI 1.3–2.6), and readmission (OR 1.7; 95% CI 1.2–2.5). We found no associations between undernutrition or falls and any of the secondary outcomes.

Because undernutrition was not associated with survival or complications, we assessed these outcomes using a risk score where we omitted undernutrition. The results can be found in “Appendix 3”. The HR for OS increased for score 1 (HR 2.5; 95% CI 1.4–4.6), 2 (HR 4.7; 95% CI 2.2–10.4), and 3 (HR 15.1; 95% CI 6.1–37.4). A score of 1 was associated with any complication (OR 1.8; 95% CI 1.2–2.9); the other scores were not. Mixed results were seen for the other outcomes.

## Discussion

A risk sumscore that reflects the cumulative risk of four geriatric domains (delirium, undernutrition, falls, and physical impairment) in older colorectal cancer patients was shown to be highly prognostic for mortality and morbidity after colorectal cancer surgery. In this study, patients with high risk sumscore had greatly increased hazard for mortality and complications independently of age and ASA score. Almost half of patients with high risk sumscore died within 3 years after surgery.

This study shows that this easy-to-use and well-implemented tool, which is aimed to direct geriatric care interventions, can also provide insight into individual risks of morbidity and mortality after colorectal cancer surgery in older patients and hence provide opportunities to discuss outcomes of treatment and shared decision-making.

Two prior studies have been performed on the VMS geriatric domains. Heim et al. included more than 800 acute or electively hospitalized patients and showed that impairment in three or more domains was strongly associated with functional decline, death, and high healthcare demand up to 3 months after hospitalization. The separate domains in that study did not satisfactorily predict the incidence of these adverse outcomes, as found in the current study for the risk for undernutrition.^15^ In addition, this adapted risk score of Heim, where patients aged 70–80 years are considered at risk when positive on three or more of the four VMS domains, appeared not useful for this study. Only 12 out of 356 patients (5%) under 80 years old in our study would be identified as at risk, missing important prognostic information for the majority of patients, most likely caused by differences between the populations of these two studies.

Similar to Heim and in a similar population, Oud et al. found an incremental risk for 6-month mortality when more domains were impaired.^16^ The results of the current study confirm these results for patients admitted for elective colorectal cancer surgery and show a sustained mortality risk beyond the first year.

As stated above, we found no association between risk for undernutrition and survival and complications, even though undernutrition is an acknowledged risk factor for complications in abdominal surgery.^17^–^19^ This may be related to the tools used to detect undernutrition. Multiple screening tools have been proposed by the European Society for Clinical Nutrition and Metabolism (ESPEN).^20^ However, all proposed tools differ in sensitivity and positive predictive value for adverse outcome, and the SNAQ and MUST screening tools show somewhat worse performance in this field compared with the more comprehensive NRS 2002.^21^ In addition, two categories of patients with high risk both for undernutrition and for complications and death were excluded from the present analysis: stage IV colorectal cancer patients and patients with acute or emergency indications for surgery. Of note, when undernutrition was omitted from the risk score, the HR for OS increased for survival, but the risk scores were no longer prognostic for any complication, possibly due to a modifying effect of undernutrition on the other domains.

For delirium, several preoperative risk factors have been reported, including advanced age, cognitive impairment, earlier delirium, and functional dependency.^22^ In this study, there was no objective assessment of cognitive function. Although the three-item delirium risk assessment has not yet been validated, it is promising that these three questions were also associated with postoperative delirium. When interested in cognitive function, other tools, such as MMSE,^23^ would be appropriate.

Strengths of our study include its multicenter design, respectable sample size, and the completeness of data. This study also has several limitations. First, we chose to include only patients with elective surgery. This may be a missed opportunity to obtain additional prognostic information and improve treatment decisions for patients in the emergency setting, who are especially at risk for complications and mortality.^24^,^25^ Second, patients were selected from the surgical audit, hence a decision to operate had been made. This introduced a possible selection bias, with patients highly dependent on care not being included in our analysis. The inability to report on preoperative instrumental ADL functioning (iADL) and iADL/ADL functioning as outcome is another limitation. It can be argued that, in addition to survival and complications, maintaining independence is a very relevant outcome after cancer surgery for older patients.^26^ Furthermore, the magnitude of the impact of preoperative impairments on adverse postoperative outcomes might have weakened given the intervention attached to the risk scores. Lastly, we note that this tool could be used to discuss outcomes of treatment and shared decision-making but does not replace a GA.

The older colorectal cancer population is growing, thus it is important to identify patients at risk of unfavorable outcomes. In addition, the colorectal cancer screening programs that have been introduced in recent years will increase the number of older patients with low stages of disease for whom surgical risk and cancer risk must be carefully weighed. Colorectal cancer surgery is now considered generally safe in older patients,^27^ with decreasing mortality rates over the past decades, but morbidity and mortality rates are still higher compared with the younger population.^28^ As the risk assessment tools used in our study have already been successfully introduced in many Dutch hospitals, the cumulative risk sumscore can provide valuable information, which can be used in shared decision-making with patients regarding their prognosis and treatment.

## Conclusions

A geriatric sumscore that reflects an individual’s risk for delirium, undernutrition, falls, and physical impairment has strong predictive value for morbidity and mortality after colorectal cancer surgery in older patients. This information can be used in shared decision-making and may be included in risk models for morbidity and mortality in older colorectal cancer patients.

## Appendix 2: Complications and Outcomes Stratified Based on the Risk Sumscore

**Table:**

	Low risk	Intermediate risk	High risk	
	Sumscore 0	Sumscore 1-2	Sumscore 3-4	
	n = 303	n = 220	n = 27	*p* value
Any complication	87 (29)	90 (41)	14 (52)	0.002
Any Surgical complication	43 (14)	41 (19)	4 (15)	0.4
Need for reintervention	22 (7)	26 (12)	2 (7)	0.5
Anastomotic leakeage	7 (2)	14 (6)	1 (4)	0.07
Non-surgical complication	44 (15)	49 (22)	10 (37)	0.004
Cardiopulmonary complication	20 (7)	22 (10)	6 (22)	0.02
Delirium	11 (4)	20 (9)	3 (11)	0.02
Discharge not to home	31 (10)	59 (27)	8 (30)	< 0.001
Readmission	21 (7)	23 (10)	4 (15)	0.2
30-day mortality	6 (2)	3 (1)	5 (19)	<0.001
Six months mortality	10 (3)	8 (4)	7 (26)	< 0.001
One year mortality	11 (4)	13(6)	7 (26)	< 0.001

Values are expressed in numbers with percentages,  %. p-value for difference between groups

## Appendix 3: Multivariate Analysis Postoperative Outcomes for Risk Score with and Without Undernutrition

**Table:**

Outcome	Overall survival	Any complication	Any surgical complication	Non-surgical complication	Cardiopulmonary complications	Delirium	Discharge not to home	Readmission < 30 days
Risk sumscore including undernutrition								
Score 0		Reference	Reference	Reference	Reference	Reference	Reference	Reference
Score 1–2	**1.9 (1.1–3.5)**	**1.8 (1.2–2.7)**	1.5 (0.9–2.5)	1.3 (0.8–2.2)	1.4 (0.7–2.7)	**2.9 (1.3–6.4)**	**2.7 (1.6.5)**	1.7 (0.9–3.0)
Score 3–4	**8.7 (4.0–19.2)**	**2.4 (1.02–5.5)**	1.0 (0.3–3.3)	**3.1 (1.2–7.7)**	2.2 (0.7–6.9)	2.5 (0.6–10.4)	2.5 (0.9–6.9)	2.1 (06–7.0)
Risk sumscore without undernutrition								
Score 0	Reference	Reference	Reference	Reference	Reference	Reference	Reference	Reference
Score 1	**2.5 (1.4–4.6)**	**1.8 (1.2–2.9)**	**1.9 (1.1–3.3)**	1.3 (0.8–2.3)	1.8 (0.9–3.7)	**4.0 (1.8–8.9)**	**2.7 (1.5–4.6)**	**2.1 (1.1–4.3)**
Score 2	**4.7 (2.2–10.4)**	2.1 (1.0–4.2)	0.9 (0.3–2.6)	**2.6 (1.2–5.6)**	1.8 (0.6–5.2)	1.9 (0.5–7.5)	**3.7 (1.6–8.5)**	1.6 (0.5–5.0)
Score 3	**15.1 (6.1–37.4)**	3.2 (1.0–10.8)	1.8 (0.5–7.5)	2.6 (0.8–8.8)	3.6 (0.9–13.9)	6.2 (1.4–27.9)	3.3 (0.9–12.0)	3.4 (0.8–14.5)

Values are expressed as indicated in OR with 95% Confidence Interval. Significant values (p < 0.05) are presented in bold. Mutivariate model include: age, gender, tumor stage, and ASA score
